# Online Health Information Seeking and eHealth Literacy Among Patients Attending a Primary Care Clinic in Hong Kong: A Cross-Sectional Survey

**DOI:** 10.2196/10831

**Published:** 2019-03-27

**Authors:** David Ka-Ki Wong, Man-Kuen Cheung

**Affiliations:** 1 University Health Service The University of Hong Kong Pokfulam China (Hong Kong)

**Keywords:** online health information seeking, eHealth literacy, primary care, Hong Kong

## Abstract

**Background:**

Previous studies have suggested that patients’ online health information seeking affects their medical consultations and patient-doctor relationships. An up-to-date picture of patients’ online health information-seeking behaviors can inform and prepare frontline health care professionals to collaborate, facilitate, or empower their patients to access and manage health information found online.

**Objective:**

This study explores the prevalence, patterns, and predictors of online health information-seeking behaviors among primary care patients in Hong Kong, and the relations between online health information seeking and electronic health (eHealth) literacy.

**Methods:**

Patients attending a university primary care clinic in Hong Kong were asked to complete a questionnaire survey on their demographic backgrounds; health status; frequency and pattern of online health information seeking; contents, sources, and reasons for online health information seeking; and their eHealth literacy. eHealth literacy was measured by the validated eHealth Literacy Scale (eHEALS). Regression analyses explored various demographic and behavioral predictors to online health information seeking, and predictors to eHealth literacy.

**Results:**

In all, 97.32% (1162/1194) respondents used the internet, of which 87.44% (1016/1162) had used the internet to find health information. Most respondents (65.97%, 665/1008) searched once monthly or more. Few (26.88%, 271/1008) asked their doctor about health information found online, but most doctors (56.1%, 152/271) showed little or no interest at all. The most sought topic was symptom (81.59%, 829/1016), the top reason was noticing new symptoms or change in health (70.08%, 712/1016), the most popular source was online encyclopedia (69.98%, 711/1016), and the top reason for choosing a source was convenience (55.41%, 563/1016). Poisson regression analysis identified high eHEALS score, fair or poor self-rated health, having a chronic medical condition, and using the internet several times a day as significant predictors of online health information seeking. Multiple regression analysis identified lower age, better self-rated health, more frequent internet use, more frequent online health information seeking, and more types of health information sought as significant predictors to higher eHealth literacy.

**Conclusions:**

Online health information seeking is prevalent among primary care patients in Hong Kong, but only a minority shared the information with doctors. Websites were chosen more for convenience than for accuracy or authoritativeness. Doctors should recognize patients’ online health information-seeking behavior, and facilitate and empower them to search for high-quality online health information.

## Introduction

The rapid development of the internet has significantly changed the way people obtain information. Now a vast amount of information can be accessed instantly. In the health care domain, the internet has changed the way people find and receive health information, from passive information received from doctors’ advice and the mass media to active information sought through Web searches (asking “Dr Google” [1]). Sources of information have also evolved from static and authoritative sources such as books and printed journals to the more dynamic and user-contributed contents such as blogs, online forums, and social networking sites [2-4].

Online health information seeking by patients has been shown to affect medical consultations and patient-doctor relationships [5-8]. Patients increasingly turn to the internet to prepare for doctors’ consultations, to discuss information they found online with their doctors, or to complement, validate, and challenge the information offered by doctors [7,9-11]. Various predictors, such as age, gender, education, socioeconomic status, health status, and internet usage, were shown to affect the prevalence and extent of online health information seeking in previous studies [10-14]. The Pew Internet and American Life Project has shown that female gender, young age, and high education level were associated with more online health information seeking [12]; whereas, in a survey of primary care patients in Scotland, employment status, educational attainment, geographic location, age, and gender were associated with online health information seeking [10].

A bibliometric study in 2015 identified 533 publications on the topic of online health information seeking [15]. Although the topic has become an increasingly important research focus, the majority of the research was from the United States, and either focused on internet users in general (eg, consumers, students, parents) or patients with specific diseases (eg, human immunodeficiency virus, sexually transmitted diseases, cancer). In Hong Kong, only one exploratory study on the prevalence and patterns of online health information seeking was done in 2006; a convenience sample of 443 members of the general public was surveyed in shopping malls and subway stations, and 44% of respondents had looked for health information online in the past 6 months. Among the health surfers, the majority (78%) visited websites from the government, hospitals, or nonprofit organizations [16].

With the rapid development of the internet and its widespread use, doctors will encounter more “internet-informed” patients. Government statistics showed a 26.5% increase in the proportion of the Hong Kong population using the internet in the past decade (from 62.9% in 2006 to 89.4% in 2017) [17], and the prevalence and patterns of online health information seeking will likely have changed since the previous study by Yan [16]. This study aims to map an up-to-date picture of online health information-seeking behaviors among primary care patients in Hong Kong. The results can better inform frontline doctors, public health professionals, and health educators on this issue, and better prepare them to collaborate, facilitate, or empower patients to access and manage health information online.

The study objectives were to (1) determine the prevalence and pattern of online health information seeking, (2) explore the contents, sources, and reasons for online health information seeking, (3) explore the predictors of online health information seeking, and (4) explore the predictors of electronic health (eHealth) literacy.

## Methods

### Study Design

The study was a cross-sectional, anonymous, self-administered questionnaire survey. Consecutive sampling was used. All patients attending a university primary care clinic in Hong Kong for consultation during the data collection period (March to April 2017) were invited to complete the questionnaire. The following groups of patients were excluded from the study: (1) age younger than 16 years, (2) unable to consent (eg, mentally incapacitated), (3) unable to read or understand the questionnaire, and (4) those who had filled in the questionnaire previously.

Questionnaires were distributed to patients by nursing staff during preconsultation health assessments. Patients could opt for either the Chinese or English version of the questionnaire, which was to be filled in while awaiting consultation and returned to collection boxes in the waiting hall. The survey required approximately 5 minutes to complete.

### Study Population and Sampling

The study population was patients attending the primary care clinic of a university in Hong Kong. The clinic serves university students, staff, and their dependents, as well as certain retirees. The clinic population is approximately 47,000.

For sample size estimation, the formula for cross-sectional studies was used, where sample size  *n=* Np(1–p)/[(d^2^/Z^2^_1–α/2_*(N–1)+p(1–p)] [18]. Given a population size (*N*) of 47,000, a hypothesized proportion (*p*) of 0.5, and a margin of error (*d*) of 0.05, the minimal sample size required ranged from 382 (95% confidence level) to 655 (99% confidence level).

### Survey Instrument

The questionnaire was developed based on a review of the literature on online health information seeking and eHealth literacy. Items from previously validated instruments were included where appropriate, including one item on self-rated health status (from SF-12 version 2 health survey [19]), and the full eHealth Literacy Scale (eHEALS) [20]. The final questionnaire consisted of 25 items, covering demographic backgrounds, health status, online health information-seeking behavior, and eHealth literacy.

The eHEALS was used because it is the most widely used validated measure of eHealth literacy; it has been validated with various population groups [20-22]. eHEALS contains eight questions on a 5-point Likert scale, of which various aspects of self-perceived eHealth literacy were measured. The sum of all items is a composite measure, with high scores indicating greater literacy. Permission was obtained from the original author for reuse and translation of eHEALS in this study.

The questionnaire was reviewed by five domain experts (two family physicians, one community pharmacist, one health education nurse, and one public health researcher), and content validity of each question was rated on a 4-point Likert scale (not relevant, somewhat relevant, quite relevant, highly relevant). The item-level content validity index (CVI) was computed as the proportion of experts who rated a question as quite or highly relevant [23]. The item-level CVIs of all questions were rated 1.00, and the scale-level CVI thus computed was also 1.00.

The Chinese version was translated by the principal investigator with feedback from the domain experts; back-translation was done by a professional translator to ensure the two language versions were conceptually equivalent [24]. In this study, Cronbach alpha of the Chinese version of eHEALS was .891, and that of the English version was .918, which indicates a high level of internal consistency, and matched the Cronbach alpha of .88 in the original study [20].

The questionnaires were pilot-tested on 52 patients of different gender, age, and education level, and they were individually debriefed by the principal investigator. Some minor rewordings on the Chinese version were done based on the feedback received, and the questionnaires were finalized after a second round of back-translation (Multimedia Appendices 1 and 2).

### Statistical Analysis

Data were analyzed using IBM SPSS Statistics version 24.0 (IBM Corp, Armonk, NY, USA). Frequency tables were computed to check for completeness, range, and consistency. Descriptive statistics were computed to summarize the data, with means and standard deviations calculated where applicable. A Poisson regression analysis explored the demographic and behavioral predictors to the extent of online health information seeking. A multiple regression analysis explored the demographic and behavioral predictors to eHealth literacy. Statistical significance was established at *P*<.05 for all tests.

### Ethical Consideration

The study was an anonymous survey, with no personal information collected, and involved minimal risk. Participation was completely voluntarily; patients could refuse to participate without any negative consequences. The purpose of the study was explained in a cover letter; informed consent was implied by completing the questionnaire. Ethics approval was received from the Human Research Ethics Committee of The University of Hong Kong (ref: EA1702020) before the study commenced.

## Results

A total of 1291 questionnaires were distributed, which yielded a response rate of 94.50% (1220/1291). Of the returned questionnaires, 26 were excluded from analysis due to grossly incomplete data (eg, missing most demographic data or a whole section of questions). This sample of 1194 respondents represented 2.54% of the total clinic population (N=47,000). Overall, 91.96% (1098/1194) of respondents opted for the Chinese questionnaire.

### Demographics of Study Sample

The demographic characteristics of the study sample are listed in Table 1. Approximately 60% (717/1179) of respondents were female. The respondents spanned all age groups and occupation ranks. Approximately half of the respondents were students with a tertiary education level. Most respondents rated their own health as good (40.35%, 481/1192) or very good (29.78%, 355/1192), whereas 26.17% (312/1192) rated their health as fair or poor. In addition, 19.10% (225/1178) had chronic medical conditions requiring regular follow-up or treatment. Almost all respondents (97.32%, 1162/1194) used the internet; the majority of them used the internet several times a day (74.46%, 863/1159) and spent more than 3 hours per day on the internet (51.42%, 596/1159).

### Prevalence and Pattern of Online Health Information Seeking

Of the respondents who used the internet, 87.44% (1016/1162) had found health information online in the past (Table 2). Most of them reported a frequency of online health information seeking from once every few months (29.27%, 295/1008), once a month (16.87%, 170/1008), to several times a month (24.01%, 242/1008). Other than finding information for oneself (94.96%, 961/1012), most also searched on behalf of family members (69.17%, 700/1012), and some searched for friends and colleagues (29.25%, 296/1012). The majority used mobile phones (74.11%, 747/1008) and laptop computers (58.43%, 589/1008) for online health information seeking.

**Table 1:** Characteristics of the study sample (N=1194).

Characteristic		n (%)	Missing data, n
**Age (years)**			**5**
	16-24	572 (48.11)	
	25-34	220 (18.50)	
	35-44	143 (12.03)	
	45-54	124 (10.43)	
	≥55	130 (10.93)	
**Gender**			**15**
	Male	462 (39.19)	
	Female	717 (60.81)	
**Education level**			**6**
	Secondary and below	144 (12.12)	
	Tertiary	646 (54.38)	
	Postgraduate	398 (33.50)	
**Occupation**			**8**
	Managers, professionals, and academic staff	244 (20.57)	
	Technicians and associate professionals	70 (5.90)	
	Clerical, services, and sales workers	161 (13.58)	
	Craft workers and laborers	10 (0.84)	
	Student	640 (53.96)	
	Housewife, retired, and unemployed	61 (5.14)	
**Self-rated health status**			**2**
	Excellent	46 (3.86)	
	Very good	355 (29.78)	
	Good	481 (40.35)	
	Fair	290 (24.33)	
	Poor	20 (1.68)	
Have chronic medical condition		225 (19.10)	16
Habit of using the internet		1162 (97.32)	
**Access the internet with: ^a,b^**			
	Desktop computer	533 (45.87)	
	Laptop computer	801 (68.93)	
	Tablet	322 (27.71)	
	Mobile phone	941 (80.98)	
**Frequency of using the internet^b^**			**3**
	Several times a week or less	46 (3.97)	
	Every day	250 (21.57)	
	Several times a day	863 (74.46)	
**Time spent using the internet per day (hours)^b^**			**3**
	<1	64 (5.52)	
	1 to <2	231 (19.93)	
	2 to <3	268 (23.12)	
	3 to <4	187 (16.13)	
	≥4	409 (35.29)	
Use wearable health-monitoring devices^b^		189 (16.31)	

^a^Multiple responses allowed.

^b^Questions only for respondents who used the internet (n=1162).

**Table 2:** Prevalence and pattern of online health information seeking for patients who had used the internet to find health information (n=1016).

Characteristic		n (%)	Missing data, n
**Frequency**			**8**
	Once a year or less	48 (4.76)	
	Every few months	295 (29.27)	
	Once a month	170 (16.87)	
	Several times a month	242 (24.01)	
	Once a week	92 (9.13)	
	Several times a week	118 (11.71)	
	Every day	43 (4.27)	
**Finding information for:^a^**			**4**
	Myself	961 (94.96)	
	Family members	700 (69.17)	
	Friends or coworkers	296 (29.25)	
**Devices used^a^**			**8**
	Desktop	410 (40.67)	
	Laptop	589 (58.43)	
	Tablet	192 (19.05)	
	Mobile phone	747 (74.11)	
Asked doctor about online health information		271 (26.88)	8
Shared online health information with doctor^b^		124 (45.8)	
Asked about specific disease^b^		222 (81.9)	
Asked for specific treatment, test, or referral^b^		143 (52.8)	
**Doctors’ interest about online health information^b^**			
	Very interested	6 (2.2)	
	Quite interested	47 (17.3)	
	Slightly interested	106 (39.1)	
	Not at all interested	46 (17.0)	
	Don’t know or can’t remember	66 (24.4)	

^a^Multiple responses allowed.

^b^Questions for respondents who asked a doctor about online health information only (n=271).

Only a minority of respondents (26.88%, 271/1008) had ever asked a doctor about health information they found online. Of those who asked, 45.8% (124/271) shared the information with the doctor through email, printout, or mobile phone screenshots. The majority of them asked about a specific disease (81.9%, 222/271), or specific treatment, test, or referral (52.8%, 143/271). When asked about health information found online, the perceived responses from doctors were uninspiring: 39.1% (106/271) of doctors were only slightly interested, and 17.0% (46/271) were not at all interested about the health information respondents found online.

### Contents, Sources, and Reasons for Online Health Information Seeking

A variety of online health information was sought by the respondents (Table 3). The top three types of information sought were symptoms (81.59%, 829/1016), a disease or condition (70.47%, 716/1016), and medication (57.19%, 581/1016). A total of 44.49% (452/1016) of respondents sought information on healthy behaviors. A minority of respondents sought information on Chinese medicine (20.77%, 211/1016), alternative medicine (11.52%, 117/1016), and health insurance (10.93%, 111/1016). On average, 4.36 types of information were sought per respondent (SD 2.11).

Regarding the reasons for seeking health information online, the majority of respondents cited noticing new symptoms or a change in health (70.08%, 712/1016), for knowledge or curiosity (51.57%, 524/1016), and deciding to change behaviors or daily routine (50.98%, 518/1016). Less than a quarter of respondents sought information to prepare for a doctor’s consultation (23.62%, 240/1016); being prescribed a new medication, test, or treatment (21.65%, 220/1016); being diagnosed a new medical condition (19.98%, 203/1016); or having doubts about information given by doctor (11.42%, 116/1016).

For the source of health information, most respondents consulted online encyclopedias (eg, Wikipedia; 69.98%, 711/1016), health portals or medical encyclopedias (eg, MIMS, MedlinePlus, WebMD; 41.83%, 425/1016), or Q&A sites (eg, Yahoo! Answers, Baidu Knows; 40.85%, 415/1016). Official websites were less consulted: 36.61% (372/1016) visited hospital or clinic websites, 34.55% (351/1016) visited government websites, 27.85% (283/1016) visited university websites, and 26.18% (266/1016) consulted nonprofit organization websites. The mean number of sources sought per respondent was 4.22 (SD 2.35).

Convenience was the top reason (55.41%, 563/1016) for choosing a particular website for health information, followed by easy to understand (51.97%, 528/1016) and top results from search engines (41.14%, 418/1016); only a minority of respondents cited recommendations from health care professionals (15.26%, 155/1016) or family and friends (9.35%, 95/1016) as the reason for choosing a website.

**Table 3:** Contents, sources, and reasons for online health information seeking (n=1016).

Question		n (%)
**Online health information sought^a^**		
	Symptom	829 (81.59)
	Disease or condition	716 (70.47)
	Medication	581 (57.19)
	Service info	470 (46.26)
	Healthy behaviors	452 (44.49)
	Treatment and procedure	367 (36.12)
	Tests and investigations	288 (28.35)
	Vitamins and supplements	282 (27.76)
	Chinese medicine	211 (20.77)
	Alternative medicine	117 (11.52)
	Health insurance	111 (10.93)
**Reason for seeking health information online^a^**		
	Noticing new symptoms or change in health	712 (70.08)
	For knowledge or curiosity	524 (51.57)
	Deciding to change behaviors or daily routine	518 (50.98)
	Hearing or seeing something in the news wanted to learn more about	446 (43.90)
	Finding or selecting a doctor or health facility	342 (33.66)
	Dealing with an ongoing medical condition	243 (23.92)
	Preparing for a doctor’s consultation	240 (23.62)
	Being prescribed with a new medication, test, or treatment	220 (21.65)
	Being diagnosed with a new medical condition	203 (19.98)
	Having doubts about information given by doctor	116 (11.42)
**Source of online health information^a^**		
	Online encyclopedia (eg, Wikipedia)	711 (69.98)
	Health portal and medical encyclopedia (eg, MIMS, MedlinePlus, WebMD)	425 (41.83)
	Q&A sites (eg, Yahoo! Answers, Baidu Knows)	415 (40.85)
	Hospital or clinic	372 (36.61)
	Government	351 (34.55)
	News sites	317 (31.20)
	Internet forums and message boards	295 (29.04)
	University	283 (27.85)
	Nonprofit organization	266 (26.18)
	Social media (eg, Facebook, Twitter)	259 (25.49)
	Video-sharing sites (eg, YouTube)	248 (24.41)
	Commercial sites	159 (15.65)
	Blogs	155 (15.26)
**Reason for choosing the source^a^**		
	Convenience	563 (55.41)
	Easy to understand	528 (51.97)
	Top results from search engines	418 (41.14)
	I think it’s trustworthy	323 (31.79)
	Usual habit	303 (29.82)
	Recommended by professionals	155 (15.26)
	Recommended by family or friends	95 (9.35)

^a^Multiple responses allowed.

### Predictors to Online Health Information Seeking

Prior to regression analyses, missing values analysis was performed. Overall, only 0.422% of items were missing from the dataset. Missing data were shown to be missing completely at random (MCAR) as evidenced by a nonsignificant Little MCAR test (*P*=.72). Single imputation of the missing values was performed using the expectation-maximization algorithm.

A Poisson regression using a logarithmic link function was performed to explore various demographic and behavioral factors that impact the extent of online health information seeking (Table 4). The number of types of health information sought (online health information-seeking info score) was chosen to represent the extent of online health information seeking as supported by previous research [10]. Poisson regression was used because the online health information-seeking info score is a count variable. After adjusting for other covariates, only fair or poor health status, having a chronic medical condition, using the internet several times a day, and a higher eHEALS score were significant positive predictors of online health information seeking (df=992, log likelihood=–2109.024).

### Predictors of eHealth Literacy

A multiple regression analysis was performed to explore the various demographic and behavioral factors that impact eHealth literacy (Table 5). The regression model was statistically significant and accounted for 11.5% of eHealth literacy (*R*^2^=.126, adjusted *R*^2^=.115, *F*_12,1003_=12.038, *P*<.001). After adjusting for other covariates, more frequent internet use, more frequent online health information seeking, and more types of health information sought were significant positive predictors of eHealth literacy, whereas age and poorer health status were significant negative predictors.

**Table 4:** Summary of Poisson regression analysis for variables predicting online health information seeking (n=1016).

Variable		Regression coefficient (SE)	Wald χ^2^_1_	*P* value	AOR^a^ (95% CI)
(Intercept)		0.557 (0.187)			
**Age (years)**					
	16-24 (Ref^b^)				
	25-34	0.006 (0.057)	0.013	.91	1.006 (0.899-1.126)
	35-44	0.063 (0.076)	0.686	.41	1.065 (0.917-1.237)
	45-54	0.022 (0.081)	0.074	.79	1.022 (0.872-1.199)
	≥55	0.041 (0.087)	0.227	.63	1.042 (0.879-1.236)
**Gender**					
	Male (Ref)				
	Female	0.045 (0.033)	1.913	.17	1.046 (0.981-1.116)
**Education level**					
	Secondary and below (Ref)				
	Tertiary	0.060 (0.065)	0.874	.35	1.062 (0.936-1.205)
	Postgraduate	0.064 (0.070)	0.836	.36	1.066 (0.930-1.222)
**Occupation**					
	Managers, executives, and officials (Ref)				
	Professionals and academic staff	0.106 (0.077)	1.923	.17	1.112 (0.957-1.292)
	Technicians and associate professionals	–0.048 (0.092)	0.275	.60	0.953 (0.795-1.142)
	Clerical and office workers	–0.013 (0.084)	0.026	.87	0.987 (0.837-1.163)
	Other workers or not currently employed	–0.150 (0.101)	2.212	.14	0.861 (0.707-1.049)
	Student	–0.011 (0.089)	0.016	.90	0.989 (0.831-1.177)
**Self-rated health status**					
	Excellent, very good, or good (Ref)				
	Fair or poor	0.151 (0.035)	18.510	<.001	1.163 (1.086-1.247)
Have chronic medical condition		0.088 (0.040)	4.790	.03	1.092 (1.009-1.181)
**Frequency of using the Internet**					
	Several times a week or less (Ref)				
	Every day	0.172 (0.101)	2.903	.09	1.188 (0.974-1.448)
	Several times a day	0.208 (0.099)	4.399	.04	1.231 (1.014-1.496)
**Time using the internet per day (hours)**					
	<1 (Ref)				
	1 to <2	0.020 (0.084)	0.059	.81	1.021 (0.866-1.204)
	2 to <3	0.020 (0.085)	0.056	.81	1.020 (0.863-1.206)
	3 to <4	0.092 (0.088)	1.094	.30	1.097 (0.922-1.304)
	≥4	0.083 (0.084)	0.981	.32	1.087 (0.922-1.282)
Uses wearable health-monitoring devices		0.037 (0.040)	0.872	.35	1.038 (0.960-1.121)
eHEALS^c^ score		0.018 (0.004)	25.796	<.001	1.018 (1.011-1.025)

^a^AOR: adjusted odds ratio.

^b^Ref: reference group.

^c^eHEALS: eHealth Literacy Scale.

**Table 5:** Summary of multiple regression analysis for variables predicting eHealth literacy (n=1016).

Variable	Unstandardized regression coefficient (SE)	Standardized regression coefficient	*t*_1003_	*P* value
(Intercept)	27.188 (1.564)			
Age	–0.484 (0.149)	–0.145	–3.249	<.001
Gender (male=0)	–0.450 (0.286)	–0.047	–1.574	.12
Education level	–0.011 (0.246)	–0.002	–0.046	.96
Occupation	–0.184 (0.111)	–0.070	–1.656	.10
Self-rated health status (excellent to poor)	–0.918 (0.187)	–0.153	–4.919	<.001
Have chronic medical condition	0.645 (0.374)	0.056	1.726	.09
Frequency of internet use	0.849 (0.286)	0.096	2.967	.003
Hours of internet use per day	–0.008 (0.116)	–0.002	–0.070	.94
Use wearable health-monitoring devices	0.393 (0.362)	0.033	1.087	.28
Frequency of online health information seeking	0.493 (0.093)	0.170	5.328	<.001
Number of types of health information sought	0.230 (0.081)	0.106	2.831	.01
Number of sources of health information sought	0.038 (0.073)	0.019	0.521	.60

## Discussion

### Principal Findings

Results from this study suggest that among the study population, consisting mostly of university students and staff, internet use is extensive and online health information seeking is highly prevalent. For those who did seek health information online, most searched at least once every few months, for themselves as well as for family and friends. The mobile phone was the most used device to access the internet in general as well as for seeking online health information, which implies a desire for instant information. Rates of online health information seeking are much more prevalent compared to a local study done a decade ago [16], but our figures are comparable to more recent studies done overseas [4,10,25].

In contrast with the high prevalence of online health information seeking, fewer respondents asked their doctors about the health information they found online. This could be due to a perceived lack of interest from the doctors; indeed, for most of those who did ask, their doctors were apparently not interested. This could have led to a vicious cycle of poor patient-doctor communication.

A large variety of health information was sought for different reasons, but the vast majority of respondents searched for symptoms and diseases, for preconsultation self-diagnosis, and to decide whether to consult a doctor or not. Approximately half of the respondents sought information about healthy behaviors and were deciding on changing their daily habits, which indicates health consciousness. Few respondents searched for information postconsultation or when given a new diagnosis, medication, or treatment. Even fewer searched because of doubts about doctors’ information—this might be explained by a strong patient-doctor trust or less desire to challenge the doctors’ authority.

Compared to the previous local study [16], fewer respondents sought health information at official websites such as hospitals, government, universities, or nonprofit organizations. Coupled with the finding that the top reasons for choosing a website were convenience and that it was easy to understand, this suggests that the respondents were less concerned about the accuracy or quality of health information online, or unaware of the possible consequences of receiving inaccurate health information online.

Fair or poor health status and having a chronic medical condition were found to be predictors of online health information seeking, which implies that online health information seeking is need-based. The eHEALS score was also found to be a significant predictor, which is consistent with previous studies [11,14], suggesting that eHealth literacy serves as an enabler to online health information seeking. In contrast with previous studies [15,25], age, gender, education, and occupation were not shown to be predictors to online health information seeking. Although there was no significant generation gap in the extent of online health information seeking, older age was associated with lower eHealth literacy.

It is also worth noting that although patients with poorer health status tended to search for health information online more often, they also had lower eHealth literacy—this group of patients could be more vulnerable to unreliable online health information and the risk to health it entails.

### Relevance to Clinical Practice

According to McMullan [26], doctors may respond in three different ways toward patients’ online health information-seeking behaviors: (1) feel threatened and respond defensively, (2) collaborate with patients to obtain and analyze the information, and (3) guide patients to reliable health websites (ie, internet prescription). It is obvious that a defensive response is unfavorable to patient-doctor rapport; indeed, a recent study has shown that failure to acknowledge and understand patients’ online health information-seeking behaviors will become a barrier to patient-doctor communication [27]. Therefore, for better rapport and patient-doctor communication, primary care doctors should facilitate and empower patients to search for health information online correctly. Thus, doctors should also be educated about the variety of and be able to assess the quality of online health information so they can better teach their patients.

Doctors, public health professionals, and health care organizations should work together to offer high-quality health information online, to improve the ease of use and readability of health care websites, and to educate patients and the general public on the importance of assessing the quality of online health information.

### Strengths and Limitations

This is the first study exploring primary care patients’ online health information-seeking behavior in Hong Kong. It provides a comprehensive and up-to-date quantitative picture of online health information seeking for primary care doctors to understand their patients’ health information needs, which they might not disclose to doctors.

Although the study sample was representative of the whole clinic population, it is skewed toward younger and more educated patients. Thus, the study’s external validity is reduced. Moreover, self-reported health status and chronic medical conditions were used in this study, as compared to physician-reported health status (eg, by chart review), which could obtain a more accurate and detailed measure of respondents’ health status.

### Conclusions

Online health information seeking is prevalent among primary care patients in Hong Kong. Most searched for information preconsultation, but only a minority shared the information with doctors. Websites were chosen more for convenience than for accuracy or authoritativeness. Doctors should recognize patients' online health information-seeking behavior, and facilitate and empower them to search for high-quality online health information.

## Appendix

Multimedia Appendix 1 Questionnaire (English version).

Multimedia Appendix 2 Questionnaire (Chinese version).

## Abbreviations

CVI: content validity index
eHEALS: eHealth Literacy Scale
eHealth: electronic health
MCAR: missing completely at random
