# Supplementary material for: Online Health Information Seeking and eHealth Literacy Among Patients Attending a Primary Care Clinic in Hong Kong: A Cross-Sectional Survey
Source: J Med Internet Res. 2019 Mar 27;21(3):e10831. doi: 10.2196/10831 (PMC6456826; doi:10.2196/10831)
Supplement: Multimedia Appendix 1 [file jmir_v21i3e10831_app1.pdf]

March 2017

Survey on Online Health Information Seeking among Patients Attending a Primary Care Clinic in Hong Kong

Dear Patients,

I am Dr David Wong at the University Health Service, University of Hong Kong. I am conducting a research project on the online health information seeking behavior of patients and would like to invite you to participate. The aim of the study is to understand the extent and factors affecting patients using the Internet to find health-related information.

You are invited to fill in the attached questionnaire, which should take less than 10 minutes to complete. The survey is strictly anonymous and no personal identifiers will be collected. All information obtained will be used for research purposes only. This survey involves minimal risk to you. You will not receive any monetary remuneration in completing the survey, but your response will provide valuable insights for the study of online health information seeking behavior. Your participation is entirely voluntary and this will not affect the quality of care you received at our clinic. You may refuse to participate without any negative consequence.

If you have any questions about the research, please feel free to contact me at 3917 2502 or dkkw@hku.hk. This study was approved by the Human Research Ethics Committee (HREC), University of Hong Kong (Ref: EA1702020). If you have questions or concerns about your rights as a research participant, please contact HREC at 2241 5267.

The completion of this survey implies your consent to participate. If you choose to participate, please complete the attached survey and return it to the collection box or to one of our clinic staff. Your help is very much appreciated.

Yours sincerely,

Dr David K K Wong
University Health Service
The University of Hong Kong

Survey Starts on Next Page

Survey on Online Health Information Seeking

We would like to ask for your opinion and your experience of using the Internet for health-related information. There are no right or wrong answers, choose an answer that suits you the most.

Please check the appropriate answers, or write your answers in the spaces provided:

Can you tell us some basic information about yourself?

1	**Age**	❑ 16-24 ❑ 25-34 ❑ 35-44 ❑ 45-54 ❑ 55-64 ❑ ≥65
2	**Gender**	❑ Male ❑ Female
3	**Education Level**	❑ Primary or below ❑ Secondary ❑ Tertiary ❑ Post-graduate
4	**Occupation**	❑ Managers/ Executives/ Officials ❑ Professionals/ Academic staff<br>❑ Technicians/ Associate professionals ❑ Clerical/ Office workers<br>❑ Services/ Sales workers ❑ Craft and related trades workers<br>❑ Plant and machine operators/ Assemblers ❑ Other workers/ Labourers<br>❑ Student ❑ Housewife ❑ Retired / Unemployed<br>❑ Others (please specify):
5	**In general, would you say your health is…**	❑ Excellent ❑ Very good ❑ Good ❑ Fair ❑ Poor
6	**Do you have any chronic medical condition?** (that require regular follow up or treatment)	❑ Yes ❑ No

7	**Do you use the Internet?**	❑ Yes ❑ No **Thank you, this is the end of survey**
8	**Do you mostly access Internet using…** *(Please check all that apply)*	❑ Desktop computer ❑ Laptop computer ❑ Tablet ❑ Smartphone
9	**How often do you use Internet?**	❑ Once a month or less ❑ Once a week ❑ Several times a week ❑ Every day ❑ Several times a day
10	**On average, how many hours do you use Internet per day?**	❑ Less than 1hour ❑ 1-2 hours ❑ 2-3 hours ❑ 3-4 hours ❑ More than 4 hours
11	**Do you use any wearable health monitoring devices or smartphone apps?** (e.g. pedometer/ heart rate monitor/ smart bracelet/ smart watch)	❑ Yes ❑ No

12	**Have you ever used Internet to find health-related information?**	❑ Yes ❑ No **Thank you, this is the end of survey**

Continue on Next Page

13	**How often do you use Internet to find health information?**				
	❑ Once a year or less	❑ Every few months	❑ Once a month	❑ Several times a month	
	❑ Once a week	❑ Several times a week	❑ Every day		
14	**Did you find the health information for…** *(Please check all that apply)*				
		❑ Myself	❑ Family members	❑ Friends or Co-workers	
15	**Which tool did you use to look for health information online?** *(Please check all that apply)*				
	❑ Desktop computer	❑ Laptop computer	❑ Tablet	❑ Smartphone	

16 **What kind of health information have you ever found online?** *(Please check all that apply)*

- ❑ Symptom
- ❑ Disease/ Condition
- ❑ Service info (e.g. doctor, hospital)
- ❑ Medication
- ❑ Test/ Investigation
- ❑ Treatment and procedure
- ❑ Chinese medicine
- ❑ Alternative medicine
- ❑ Vitamins and supplements
- ❑ Health insurance
- ❑ Healthy behaviors (e.g. diet, exercise, quit smoking)
- ❑ Others (please specify):

17 **Why did you find health information online?** *(Please check all that apply)*

- ❑ Noticing new symptoms or change in my health (Am I ill?)
- ❑ Finding/ selecting a doctor or healthcare facility
- ❑ Preparing for a doctor's consultation/ to discuss it with my doctor
- ❑ Being diagnosed with a new medical condition
- ❑ Being prescribed with a new medication, test, or treatment
- ❑ Having doubts about information given by my doctor
- ❑ Dealing with an ongoing medical condition (e.g. diabetes, high blood pressure)
- ❑ Deciding to change my behaviors/ daily routine (e.g. diet, exercise, quit smoking)
- ❑ Hearing or seeing something in the news that you wanted to learn more about
- ❑ For knowledge or curiosity
- ❑ Others (please specify):

18 **On which websites did you find the health information?** *(Please check all that apply)*

- ❑ University
- ❑ Online encyclopedia (e.g. Wikipedia)
- ❑ Government
- ❑ Q&A site (e.g. Yahoo! Answers, Baidu Knows)
- ❑ Hospital/ clinic
- ❑ Internet forum/ message board
- ❑ Non-profit organization
- ❑ Social media (e.g. Facebook, Twitter)
- ❑ Commercial site (e.g. drug/ formula milk/ personal care products manufacturer)
- ❑ Health portal/ medical encyclopedia (e.g. MIMS, PubMed, MedlinePlus, WebMD)
- ❑ News site
- ❑ Video-sharing site (e.g. YouTube)
- ❑ Blog
- ❑ Others (please specify):

Continue on Next Page

19 **Why did you choose those websites to find the health information?** *(Please check all that apply)*

❑ Recommended by professionals (e.g. healthcare professionals, dietitians) ❑ Easy to understand ❑ Usual habit

❑ Recommended by family or friends ❑ I think it's trustworthy ❑ Convenience

❑ Top results from search engines ❑ Others (please specify):

20 **Did you ever ask or discuss with your doctors about the health information found online?** ❑ Yes ❑ No **Please go to Question 25**

21 **Did you ever share with your doctors health information found online?** (e.g. by email/ print-out/ photos or screenshot on smartphone) ❑ Yes ❑ No

22 **Did you ever ask or discuss with your doctors about specific disease or diagnosis because of the health information found online?** ❑ Yes ❑ No

23 **Did you ever ask or discuss with your doctors about specific treatment, tests or referral because of the health information found online?** ❑ Yes ❑ No

24 **Were your doctors interested in hearing about the health information you found online?**

❑ Very interested ❑ Quite interested ❑ Slightly interested ❑ Not at all interested

❑ Don't know/ Can't remember

25 **For the following statements, please check the responses that best reflect your opinion and experience <u>now</u>.**

	Strongly disagree	Disagree	Undecided	Agree	Strongly agree
(a) I know **what** health resources are available on the Internet.	❑	❑	❑	❑	❑
(b) I know **where** to find helpful health resources on the Internet.	❑	❑	❑	❑	❑
(c) I know **how** to find helpful health resources on the Internet.	❑	❑	❑	❑	❑
(d) I know **how to use** the Internet to answer my questions about health.	❑	❑	❑	❑	❑
(e) I know how to use **the health information** I find on the Internet to help me.	❑	❑	❑	❑	❑
(f) I have the skills I need to **evaluate** the health resources I find on the Internet.	❑	❑	❑	❑	❑
(g) I can tell **high quality** health resources from **low quality** health resources on the Internet.	❑	❑	❑	❑	❑
(h) I feel **confident** in using information from the Internet to make health decisions.	❑	❑	❑	❑	❑

This is the end of survey. Thank you very much for your participation!

Ver. 20170317
