# Supplementary material for: Online Health Information Seeking and eHealth Literacy Among Patients Attending a Primary Care Clinic in Hong Kong: A Cross-Sectional Survey
Source: J Med Internet Res. 2019 Mar 27;21(3):e10831. doi: 10.2196/10831 (PMC6456826; doi:10.2196/10831)
Supplement: Multimedia Appendix 2 [file jmir_v21i3e10831_app2.pdf]

2017 年 3 月

病人網絡健康資訊搜尋行為研究

香港大學醫療保健處邀請您參與由王家祺醫生主理的研究調查。這是一項關於病人網絡健康資訊搜尋行為的學術研究，旨在探討病人使用互聯網尋找健康資訊的程度和相關因素。

我們邀請您完成附上的問卷，需時約十分鐘。問卷是匿名的，不會收集任何個人資料。所有收集的資料將只用於研究用途。是次研究對您的風險極低。參與本研究將不會為您帶來實質的利益，然而您的參與將對研究網絡健康資訊搜尋行為提供寶貴的資料。您的參與是完全自願性質，您可以拒絕參與是項研究，對您在本診所接受的服務不會有任何影響。

如您對是項研究有任何查詢，歡迎與研究負責人王家祺醫生聯絡（電話 3917 2502 / 電郵 dkkw@hku.hk）。如您想知道更多有關研究參與者的權益，請聯絡香港大學研究操守委員會（參考編號：EA1702020），電話 2241 5267。

完成本問卷表示您同意參與是項研究。如果您選擇參加，請填寫附上的問卷，並將其交回收集箱或診所的工作人員。 非常感謝您的支持與參與！

王家祺醫生
香港大學醫療保健處

問卷在下一頁開始

網絡健康資訊搜尋行為問卷

我們想了解您使用互聯網獲取健康相關資訊的經驗和意見。不存在絕對正確或錯誤的答案，您只需選填最能形容您本人的答案。

請圈出相應的答案，或在提供的位置填寫您的答案：

您能告訴我們一些關於您的基本資料嗎？

1 **年齡** ❑ 16-24 ❑ 25-34 ❑ 35-44 ❑ 45-54 ❑ 55-64 ❑ ≥65

2 **性別** ❑ 男 ❑ 女

3 **教育程度** ❑ 小學或以下 ❑中學 ❑ 大專 ❑ 碩士或以上

4 **職業**
❑ 經理/行政人員/官員 ❑ 專業人員/教職員
❑ 技術人員/輔助專業人員 ❑ 文職/辦公室員人員
❑ 服務/銷售人員 ❑ 工藝及相關技工
❑ 機台及機器操作員/裝配員 ❑ 工人/勞工
❑ 學生 ❑ 家庭主婦 ❑ 退休人仕/待業
❑ 其他（請說明）：

5 **總括來說，您認為您的健康狀況是...**
❑ 極好 ❑ 很好 ❑ 好 ❑ 一般 ❑ 差

6 **您患有任何慢性疾病嗎？**（需要定期覆診或治療） ❑ 有 ❑ 沒有

7 **您有使用互聯網服務（上網）的習慣嗎？** ❑ 有 ❑ 沒有
問卷完
感謝您的參與

8 **您通常使用甚麼電子工具上網？***（請選取所有適用的答案）*
❑ 桌上電腦 ❑ 手提電腦 ❑ 平板電腦 ❑ 智能手機

9 **您上網的頻密程度為？** ❑ 每月一次或更少 ❑ 每週一次
❑ 每週數次 ❑ 每天 ❑ 每天數次

10 **您平均每天花多少時間上網？**
❑ 少於一小時 ❑ 1-2 小時 ❑ 2-3 小時 ❑ 3-4 小時 ❑ 多於 4 小時

11 **您有使用任何可穿戴（可以掛在身上）的健康監控設備或智能手機應用程式嗎？** ❑ 有 ❑ 沒有
（例如： 計步器/心率監測器/智能手環/智能手錶）

12 **您曾經上網搜尋健康相關的資訊嗎？** ❑ 有 ❑ 沒有
問卷完
感謝您的參與

請轉到下一頁

13 **您有幾經常上網搜尋健康資訊？**

❑ 一年一次或更少 ❑ 每幾個月一次 ❑ 每月一次 ❑ 每月數次

❑ 每週一次 ❑ 每週數次 ❑ 每天

14 **您為誰搜尋健康資訊？***（請選取所有適用的答案）*

❑ 自己 ❑ 家人 ❑ 朋友或同事

15 **您用甚麼工具上網搜尋健康資訊？***（請選取所有適用的答案）*

❑ 桌上電腦 ❑ 手提電腦 ❑ 平板電腦 ❑ 智能手機

16 **您曾上網尋找甚麼健康資訊？***（請選取所有適用的答案）*

❑ 症狀 ❑ 疾病/病況 ❑ 醫療服務資訊（例如：醫生，醫院）

❑ 藥物 ❑ 測試/檢查 ❑ 治療/醫療程序

❑ 中醫藥 ❑ 另類療法 ❑ 維生素和保健品

❑ 醫療保險 ❑ 健康生活行為（例如：飲食，運動，戒煙）

❑ 其他（請說明）：

17 **您為什麼上網尋找健康資訊？***（請選取所有適用的答案）*

❑ 注意到新的症狀或健康變化（我生病了嗎？）

❑ 幫助尋找/選擇醫生或醫療機構

❑ 見醫生前的準備/與我的醫生討論

❑ 被診斷患有新的疾病

❑ 被處方新的藥物，測試或治療

❑ 對醫生提供的資訊有疑惑

❑ 如何控制慢性疾病（例如：糖尿病，高血壓）

❑ 希望改變我的生活習慣（例如：飲食，運動，戒煙）

❑ 在新聞中聽到或看到某些資訊，想要了解更多

❑ 知識或好奇心

❑ 其他（請說明）：

18 **您曾在哪些網站上找到健康資訊？***（請選取所有適用的答案）*

❑ 大學

❑ 政府

❑ 醫院/診所

❑ 非牟利組織

❑ 商業網站 （例如：藥物/奶粉/個人護理產品製造商）

❑ 新聞網站

❑ 網誌

❑ 網上百科全書（例如：維基百科）

❑ 問答網站（例如： Yahoo!答案/百度知道）

❑ 互聯網論壇/討論區

❑ 社交媒體（例如： Facebook / Twitter）

❑ 健康網站/醫療百科全書（例如： MIMS / PubMed / MedlinePlus / WebMD）

❑ 影片分享網站（例如： YouTube）

❑ 其他（請說明）：

請轉到下一頁

19 **您為什麼選擇那些網站來尋找健康資訊？***（請選取所有適用的答案）*

❑ 專業人員的推薦（例如醫護人員，營養師） ❑ 容易理解 ❑ 習慣

❑ 家人或朋友的推薦 ❑ 我認為該網站值得信賴 ❑ 方便

❑ 搜尋引擎排名較高的網頁 ❑ 其他（請說明）：

20 **您曾經向您的醫生詢問或討論過在網上找到的健康資訊嗎？** ❑ 有 ❑ 沒有 **請轉到第 25 條問題**

21 **您曾經把在網上找到的健康資訊與您的醫生分享嗎？** ❑ 有 ❑ 沒有

（例如：用電郵/列印本/智能手機相片或截圖）

22 **您曾經因為在網上找到的健康資訊而進一步向您的醫生詢問或討論特定疾病或診斷嗎？** ❑ 有 ❑ 沒有

23 **您曾經因為在網上找到的健康資訊而進一步向您的醫生詢問或討論特定治療，檢查或要求轉介嗎？** ❑ 有 ❑ 沒有

24 **您的醫生對您在網上找到的健康資訊是否感興趣？**

❑ 非常感興趣 ❑ 頗感興趣 ❑ 略感興趣 ❑ 完全不感興趣

❑ 不知道/不記得

---

25 **對於下列陳述，請選擇最能反映您<u>此刻</u>的意見和經歷的答案。**

	非常不同意	不同意	不確定	同意	非常同意
(a) 我知道互聯網上有**<u>甚麼</u>**健康資源	❑	❑	❑	❑	❑
(b) 我知道去**<u>那些網頁</u>**尋找有用的健康資源	❑	❑	❑	❑	❑
(c) 我知道**<u>如何</u>**在互聯網上找到有用的健康資源	❑	❑	❑	❑	❑
(d) 我知道**<u>怎樣使用</u>**互聯網來解答我的健康問題	❑	❑	❑	❑	❑
(e) 我知道怎運用互聯網上的**<u>健康資訊</u>**來幫助自己	❑	❑	❑	❑	❑
(f) 我擁有**<u>評估</u>**互聯網上健康資源質素的能力	❑	❑	❑	❑	❑
(g) 我能夠分辨出**<u>高品質</u>**與**<u>低品質</u>**的互聯網健康資源	❑	❑	❑	❑	❑
(h) 我有**<u>自信</u>**使用互聯網的資訊作出健康決策	❑	❑	❑	❑	❑

問卷完。 非常感謝您的支持與參與！

Ver. 20170317
